# Atretic preovulatory follicles could be precursors of ovarian lutein cysts in the pig

**DOI:** 10.1038/s41598-023-34563-4

**Published:** 2023-05-12

**Authors:** Adam J. Ziecik, Pawel Likszo, Jan Klos, Katarzyna Gromadzka-Hliwa, Katarzyna Knapczyk-Stwora, Olli Peltoniemi, Zdzislaw Gajewski, Monika M. Kaczmarek

**Affiliations:** 1 Department of Hormonal Action Mechanisms, Institute of Animal Reproduction and Food Research, Polish Academy of Sciences, Tuwima 10 Str., 10-747 Olsztyn, Poland; 2 Department of Endocrinology, Institute of Zoology and Biomedical Research, Jagiellonian University, Kraków, Poland; 3 Department Production Animal Medicine, Faculty of Veterinary Medicine, University of Helsinki, Helsinki, Finland; 4 Faculty of Veterinary Medicine, Center for Translational Medicine, Warsaw University of Life Science, Warsaw, Poland; 5 Molecular Biology Laboratory, Institute of Animal Reproduction and Food Research, Polish Academy of Sciences, Olsztyn, Poland

**Keywords:** Endocrinology, Reproductive biology, Animal physiology

## Abstract

Ovarian cysts contribute to reduced reproductive performance in pigs. Unfortunately, the mechanism of lutein cysts formation remains unknown. Here, we compared the endocrine and molecular milieus of intact, healthy preovulatory follicles (PF), gonadotropin (eCG/hCG)-induced healthy and atretic-like PF, as well as gonadotropin-provoked and spontaneous ovarian cysts in gilts. Several endocrine and molecular indicators and microRNA were compared in walls of PF and cysts. Intact and healthy PF, showed high estradiol/androstendione and low progesterone levels associated with CYP17A1, HSD17B1, and CYP19A1 elevation and reduced StAR/HSD3B1 protein expression. In contrast, low estradiol/androstendione and high progesterone concentrations, accompanied by decreased CYP17A1, HSD17B1, CYP19A1 and increased HSD3B1 protein abundance, appeared in atretic-like PF, gonadotropin-induced and spontaneous cysts. High progesterone receptor (PGR) protein abundance was maintained in intact and healthy PF, while it dropped in atretic-like PF, gonadotropins-induced and spontaneous cysts. The atretic PF showed high level of TNFα compared to healthy PF. In conclusion, follicular lutein cysts could be recruited from atretic-like PF with lost estrogenic milieu and inability to ovulate. Ovulatory cascade was presumably disrupted by a low PGR and high TNFα levels associated with earlier luteinization of follicular walls. These results suggest a novel mechanism of lutein ovarian cysts development in pigs and, perhaps, other species.

## Introduction

Ovarian cysts are a fertility disorder that has a profound effect on reproductive performance in the pig^1–4^. Most of the multiple large cysts results from luteinized ovarian follicles^3^. Generally, the presence of ovarian cysts leads to fewer ovulations^5^, prolonged behavioral estrus^6^, and delayed estrus to estrus interval^7,8^. Other studies indicated decreased fertility, as a measured by lower conception rates or smaller litter sizes in sows showing ovarian cysts^9,10^.

A significant negative correlation between the occurrence of ovarian cysts and the number of primary, growing and maturating ovarian follicles was found in sows^4^. Our previous results^11^ indicate a higher appearance of follicular cyst in prepubertal (66.7%) than in mature (13.9%) gilts challenged with altrenogest (an orally active progestogen) and exposed to exogenous gonadotropins. The presence of one follicular cyst is able to prevent two ovulations and consequently two corpora lutea (CLs) formation^11^, decreasing the reproductive performance of gilts.

The etiology of follicular cyst development was studied using various methods, including treatment of prepubertal gilts with antihistamine or indomethacin to limit the excessive accumulation of fluid within the follicles or suppress synthesis of PGF2α necessary for follicle rupture, respectively^12^, mechanical manipulation of ovaries followed by equine (eCG) or human (hCG) gonadotropin stimulation^13^, induction of hypo- and hyperthyroid environment in cyst-bearing gilts^5^, and administration of dexamethasone^14^. Interestingly, manipulation of the ovary during the follicular phase in cycling or prepubertal gilts resulted in the formation of cysts after eCG and hCG administration. However, cysts did not form after manipulations performed during the luteal phase. The administration of progesterone (P_4_) to mimic concentrations present in the luteal phase did not reduce the incidence of cyst formation in prepubertal gilts treated with eCG and hCG^13^.

The development of ovarian cysts seems to be linked with disturbances in the secretory function of the hypothalamic-pituitary-ovarian axis^5^ and/or with a deficiency of luteinizing hormone (LH), rather than an intrinsic ovarian abnormality^15^. Both LH and hCG activate the same receptor (LHCGR) but hCG showed greater affinity to LHCGR with a fivefold higher potency to increase cAMP production, whereas LH preferentially activate extracellular signaling, namely ERK1/2 and AKT pathways^16^. In addition, hCG has a considerably longer half-life then LH (28 h vs. 20 min, respectively) and it causes ovarian hyper stimulation syndrome^17^. Furthermore, hCG used to control follicular development in gilts and sows is able to act on hypothalamus or higher brain centers to inhibit LH release, preventing an estrogen-induced LH surge in pigs^18^. Likewise, the administration of hCG blocks the preovulatory LH surge in gilts that have been fed an orally active progesterone to synchronize estrus^18,19^. In contrast, Almond and Richards^20^ showed that tonic and pulsatile LH release was not adversely affected in sows with cystic ovaries. Other data suggest that inappropriate or inadequate production of prostaglandins during follicle rupture at ovulation may lead to the formation of ovarian cysts^21,22^.

The mechanism of follicular cyst development in pigs remains largely unknown. Two hypotheses, that (1) cysts are delivered from follicles showing reduced sensitivity to LH (measured as a reduced number of LH/hCG receptors) or that (2) insufficient synthesis or premature secretion of PGF2α is leading to the formation of ovarian cysts, have not been proved by prior studies^13^. Our recent results have indicated that exogenous gonadotropins have diverse effects on the endocrine milieu and molecular regulation of ovarian follicle development in the prepubertal and mature gilts^23,24^. Supporting the concept of the different endocrine properties of hCG and LH, a potent gestogenic and androgenic role of hCG and rather estrogenic function of LH was presented^24^. In addition, we found that reproductive maturity and altrenogest treatment have multiple effects on ovarian follicle development in gilts^23^.

We hypothesized that challenging mainly prepubertal but also a certain number of adult gilts with exogenous gonadotropins (eCG/hCG) after altrenogest priming could disturb the development of some preovulatory follicles, which would not ovulate after hCG administration (or after the native LH preovulatory surge onset) and would become ovarian cysts. To test this hypothesis, we compared the endocrine and molecular milieus of preovulatory follicles of intact gilts, experimentally induced follicles or cysts and cysts spontaneously occurring in gilts. The roles of the progesterone receptor (PGR), microRNA (miRNAs), the local regulators of steroidogenesis and tumor necrosis factor-α (TNFα) in the process of preovulatory follicle transformation into cysts were also explored.

## Materials and methods

### Selection of animals and experimental group recruitment

A pool of 15 prepubertal crossbred gilts of similar age (165 days) and weight (115 kg), without signs of estrus at approximately 180 days of age, were used. The animals were fed 20 mg daily of altrenogest (Suifertil, Medica, Poland), administered orally (5 mL) with the Suifertil pump for 18 consecutive days. The day after last day of altrenogest treatment (day 19), gilts received intramuscular injections of 1000 IU eCG (Syncrostim, Ceva Santé Animale, Libourne, France) and 72 h later were challenged with 750 IU hCG (Chorulon, Intervet International Boxmeer, Nederland). The detailed procedures are described in our previous paper^24^.

Seven gilts from this pool were ovariectomized 30 h after hCG challenge to obtain ovaries before ovulation, and their preovulatory follicles were morphologically classified as gonadotropin-induced healthy (HPF) or early atretic-like (APF) according to criteria described by Alonso-Pozos^25^ and Jolly^26^. Healthy follicles were characterized by well-vascularized follicular walls and a clarity of follicular fluid under a stereomicroscope. Early atretic-like follicles were marked out by few or no blood vessels and opalescent follicular fluid^27^. In consequence, HPF and APF follicles descended from the same gilts were included in two counterpart experimental groups (n = 6). One gilt out of 7 designated to HPF and APF follicles group ovulated before ovariectomy and was omitted from further analyses. A summary of the microscope examination of these ovaries is presented in Supplementary Table S1. The next group formed of prepubertal gilts with gonadotropin-induced postovulatory cysts (IPC; n = 8) that were ovariectomized 7 days after hCG administration. In addition, mature gilts on days 17 (17PF; n = 8) and 19-20 of the estrous cycle (19-20PF; n = 10), as well as mature gilts with spontaneously occurring cysts in mid-luteal phase of the estrous cycle (SOC; n = 10) were included in this study. Representative pictures of different kinds of ovaries with preovulatory healthy, atretic-like follicles and induced and spontaneous cysts are presented in Supplementary Fig. S1. The pigs subjected to ovariectomy were sedated with ketamine (10 mg kg^−1^), azaperon (3 mg kg^−1^), and medetomidine (0.04 mg kg^−1^). General anesthesia was maintained with isoflurane (1–2%) and propofol (2–4 mg kg^−1^). Crossbred gilts forming 17PF, 19-20PF, HPF, APF and IPC groups were from the same commercial herd and their ovaries were collected at local abattoir (17PF, 19-20PF, IPC) or after ovariectomy (HPF and APF).

All gilts bearing cysts (hormonally induced and spontaneous) had oligocystic ovaries with fewer than 10 cysts^28^ and corpora lutea with large cavity were not observed. The number of hormonally induced cysts varied from 1 to 8 on both ovaries with diameters 9–10 mm (n = 7), 11–20 mm (n = 12), and > 20 mm (n = 8). Similarly, the number of spontaneously occurring cyst varied from 1 to 7 on both ovaries and these were also categorized into three groups according to their respective diameter 9–10 mm (n = 1), 11–20 mm (n = 12) and > 20 mm (n = 16).

The experiments were performed in accordance with the national and EU guidelines for agricultural animal care (EU Directive 2010/63/UE) and the Animal Research: Reporting of in vivo Experiments (ARRIVE) guidelines for the use of animals in research, and were approved by the Local Animal Ethics Committee (University of Warmia and Mazury, Olsztyn, Poland; permission number: 38/2020).

### Sample collection

Porcine ovaries were collected from gilts during ovariectomy or after slaughtered at a local abattoir and transported to the laboratory in ice-cold phosphate-buffered saline (PBS; 137 mM NaCl; 27 mM KCl; 10 mM Na_2_HPO_4_; and 2 mM KH_2_PO_4_; pH 7.4), containing 100 IU of penicillin (Sigma-Aldrich, St. Louis, MO, USA) and 100 μg/mL of streptomycin (Sigma-Aldrich). Ovaries were placed against a ruler and photographed from different sides to count preovulatory follicles or cysts (see: “Selection of animals and experimental group recruitment”). Subsequently, follicular fluid from intact 17PF, 19-20PF, healthy (HPF), atretic-like (APF) preovulatory follicles > 6 mm and fluid from gonadotropin induced (IPC) or spontaneously occurred (SOC) cysts was aspirated with a 21 G needle and pooled. The fluid was then centrifuged at 1550×*g* for 10 min at 4 °C to remove cell debris, and stored at − 20 °C for hormones assay. Afterwards, the follicular or cyst walls were harvested by cutting out and peeling off the follicle or cyst, pooled for each animal, snap-frozen in liquid nitrogen, and kept at − 80 °C for further analysis. For each gilt, follicular/cyst fluid and walls were pooled from two to four of the largest follicles or cysts, therefore, the number of samples analyzed is the same as the number of animals.

### Histological examination of cysts

Fragments of the cyst walls were fixed in Bouin’s solution for histological examination. Fixed tissues were dehydrated in an increasing gradient of ethanol, cleared in xylene, embedded in paraplast (Sigma-Aldrich) and cut in 5-µm thick sections that were next mounted on slides coated with 3′3′-aminopropyl-triethoxysaline (Sigma-Aldrich). The slides were stained with hematoxylin QS (Vector Laboratories, Burlingame, CA, USA) for 20 s and alcoholic solution of eosin Y (Sigma-Aldrich) for 10 s. Sections were then washed in ethanol, fixed in xylene, mounted using DPX (Sigma-Aldrich) and coverslip. Histological examination was conducted to assess the type of ovarian cysts (Supplementary Fig. S2). All cysts were classified as the follicular lutein cyst^4^, including type I (granulosa lutein cells only) and type II (granulosa cells and connective tissue) follicular lutein cysts^29^.

### Prostaglandins, steroid hormones and TNFα assays

Steroid hormone concentrations in follicular or cyst fluid were determined using radioimmunoassay (RIA) kits: A4-RIA-CT for androstenedione (A_4_), E2-RIA-CT for estradiol-17-beta (E_2_), T-RIA-CT for testosterone (T), and PROG-RIA-CT for progesterone (P_4_; all from DIASource, Louvain-le-Neuve, Belgium), according to the manufacturer’s instructions. Assay sensitivity was 0.03 ng/mL for A_4_, 2.7 pg/mL for E_2_, 0.5 ng/mL for T and 0.05 ng/mL for P_4_, and intra-assay coefficients of variation were 5.9%, 10.4%, 6.5%, and 8.3%, respectively.

PGE_2_ and PGFM (13,14-dihydro-15-keto-prostaglandin F2α) concentration in follicular and cyst fluid was determined using the conventional EIA method according to Blitek et al.^30^. Anti-PGE_2_ antibodies and anti-PGFM (donated by Dr. W. Silvia, University of Kentucky, Lexington, KY, USA, Supplementary Table S2) developed in rabbits were used to determine PGE_2_ and PGFM. The sensitivity of the assay was 0.19 ng/mL for PGE_2_ and 25 ng/mL for PGFM. The intra-assay coefficients of variation were 9.4% for PGE_2_ and 12.3% for PGFM.

TNFα concentration in follicular and cyst fluid was determined using commercial TNFα Porcine Elisa Kit (Thermo Fisher Scientific, Waltham, MA, USA), according to the manufacturer’s instructions. Assay sensitivity and the intra-assay coefficients of variation were < 3 pg/mL and 6%, respectively.

### Protein extraction

The samples for Western blotting, were homogenized by sonication (Sonopuls, Bandelin Electronic GmbH & Co. KG, Berlin, Germany) on ice in lysis buffer (50 mM Tris–HCl, pH 7.4; 150 mM NaCl; 1% Triton X-100 (v/v); 0.02% sodium azide and 1 mM/L EDTA) containing 100 mM protease inhibitor cocktail (Sigma-Aldrich). Tissue homogenates were centrifuged at 800×*g* for 10 min at 4 °C and stored at − 80 °C until the analysis. The protein content was determined by Bradford method^31^.

### Western blot

Western blots were performed as previously described^24^. In brief, equal portions of protein (25 µg) from follicular or cyst walls were mixed with SDS gel-loading buffer (250 mM/L Tris–HCl, pH 6.8; 10% β-mercaptoethanol; 125 mM SDS; 40% glycerol; and 0.578 mM bromophenol blue), loaded onto a TGX Stain-Free gel (Bio-Rad, Hercules, CA, USA), electrophoresed, and subsequently transferred to a PVDF membrane (Sigma-Aldrich). Prior to the transfer of protein, the TGX gel was activated to obtain the total content of loaded protein, according to the manufacturer’s instructions. The membrane was incubated with primary antibody overnight at 4 °C, and then incubated with the corresponding secondary antibody for 1.5 h at room temperature. The primary antibodies included: anti-StAR, anti-CYP11A1, anti-HSD3B1, anti-HSD17B1, anti-CYP17A1, anti-CYP19A1, anti-PTGS2, anti-MMP2, anti-TIMP1, anti-TF, anti-VIM, anti-PGR-A/PGR-B and anti-LHCGR (donated by Dr. Marco Bonomi, Cusano Milanino MI, Italy^32^) were diluted in TBS-T buffer (Supplementary Table S2). Additional specificity tests were performed with omission of primary antibodies (Supplementary Fig. S3). Chemiluminescence was generated by using Clarity ECL substrate (Bio-Rad), developed in the ChemiDoc™ Touch Imaging System (Bio-Rad) and quantified using Image Lab 6 software (Bio-Rad). The abundance of tested proteins was quantified and normalized to the total protein content in each equivalent lane.

### Total RNA isolation and real-time PCR

Gene expression analysis was performed as previously described^24^. Briefly, total RNA was extracted from follicular or cyst walls using a mirVana microRNA Isolation Kit (Invitrogen, Thermo Fisher Scientific). Quantity and quality of the isolated RNA was checked using a NanoDrop 1000 spectrophotometer (Thermo Fisher Scientific) and Agilent Bioanalyzer 2100 (Agilent Technologies, Santa Clara, CA, USA), respectively. Reverse transcription and PCR reactions were conducted with the TaqMan RNA-to-Ct1-Step Kit (Applied Biosystems, Thermo Fisher Scientific) and TaqMan Gene Expression Assays (20×; Supplementary Table S3) on HT7900 Real-Time PCR System (Applied Biosystems). The cycle threshold (Ct) and PCR efficiency of each gene were determined using the real-time PCR Miner Software^33^. The NormFinder algorithm was used to rank the candidate reference genes (ACTB, GAPDH and HPRT1) based on their stability values^34^.

### Statistical analysis

Statistica 13 (Krakow, Poland) was used to perform the statistical analysis for (1) the content of steroid hormones and prostaglandin in the follicular and cyst fluid; (2) changes of mRNA expression in the walls of preovulatory follicles and postovulatory cysts; (3) changes of protein expression in the walls of preovulatory follicles and postovulatory cysts, and (4) correlations between studied factors (Person’s). These analyses were performed using one-way ANOVA and LSD test by selecting appropriate planned comparisons. Logarithmic transformation of data was performed for non-normal distribution. All numerical data were expressed as mean ± standard error of the mean (SEM) and differences were considered statistically significant at *p* < 0.05. Student’s t-paired test was used additionally for data obtained for preovulatory follicles (HPF and APF) derived from ovaries of the same gilts.

## Results

### Hormonal milieu of the follicular and cyst fluid

**Figure 1:**
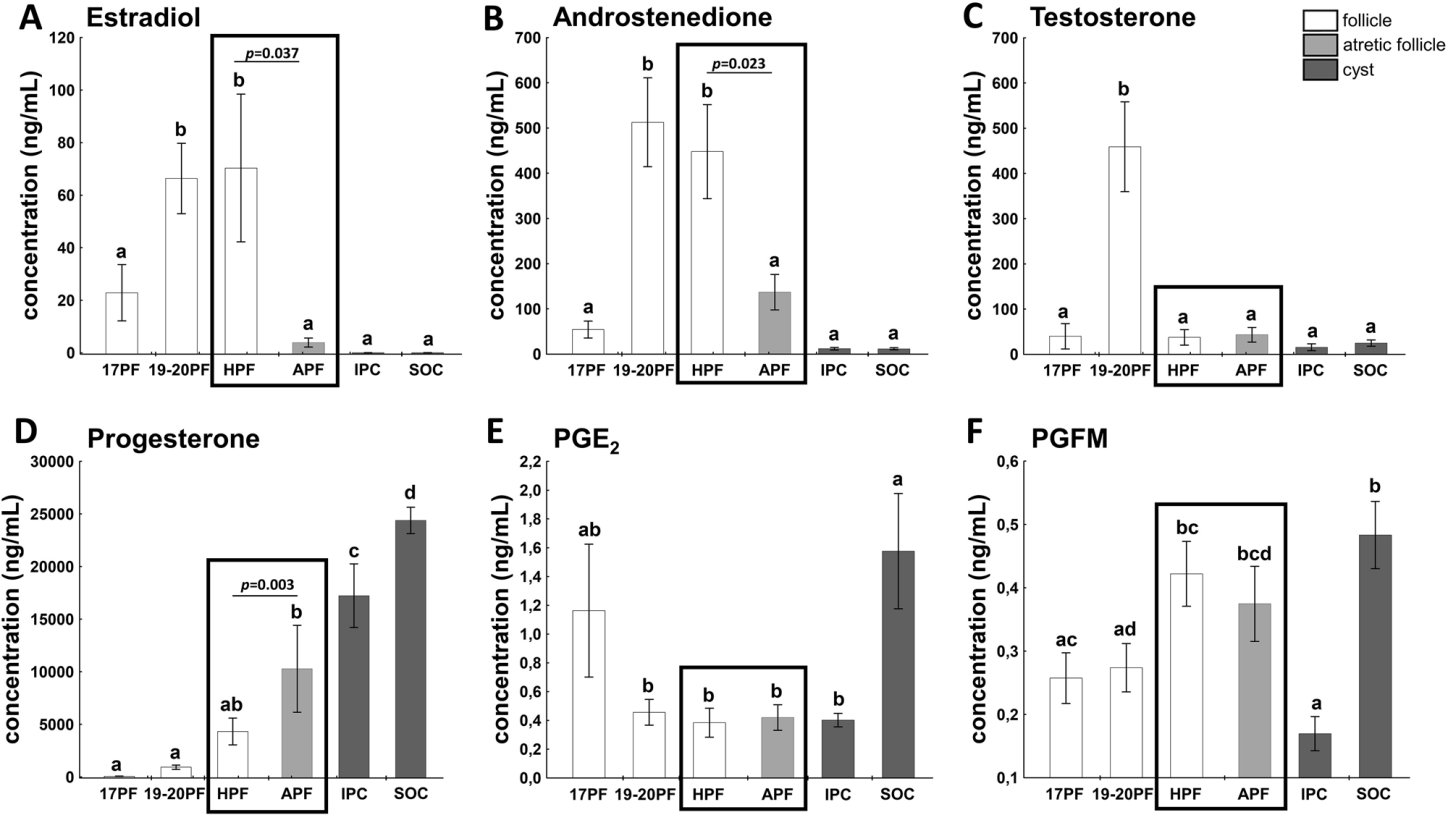
Hormonal profiles in the follicular fluid of 17PF (n = 8), 19-20PF (n = 10), HPF (n = 6) and APF (n = 6) and cystic fluid of IPC (n = 8) and SOC (n = 10). Panels show concentrations of estradiol (**A**), testosterone (**B**), androstenedione (**C**), progesterone (**D**), prostaglandin E_2_ (**E**) and prostaglandin FM (**F**). Data are presented as mean ± SEM. Data were analyzed using one-way ANOVA and LSD test by selecting appropriate planned comparisons. Letters (a, b) indicate a significant difference between groups (*p* < 0.05). For framed groups (follicles derived from ovaries of the same gilts), Student’s t-test was performed.

Concentration of E_2_ (Fig. 1A) in follicular fluid of 19-20PF and gonadotropin-induced healthy preovulatory follicles (HPF) did not differ and was 18-fold higher (*p* < 0.05) than in follicular fluid of atretic-like preovulatory follicles (APF) and about 4000-fold higher than in cystic fluid of IPC and SOC (*p* < 0.05).

Levels of A_4_ (Fig. 1B) followed the pattern of E_2_ concentration in studied groups of gilts and was the highest (*p* < 0.05) in 19-20PF, and HPF. Concentration of E_2_ and A_4_ was significantly higher in HPF than APF follicles (*p* = 0.037 and *p* = 0.023, respectively, Fig. 1A,B).

Concentration of T was the highest in 19-20PF (450 ± 99 ng/mL) and it was approximately tenfold higher than in the remaining groups (Fig. 1C).

Concentration of P_4_ gradually rose during maturation of preovulatory follicles and their transformation into cysts (Fig. 1D). Interestingly, P_4_ level was threefold higher in APF (*p* = 0.003) than in healthy counterparts (HPF). In cystic fluid of IPC and SOC levels of P_4_ were about three to fivefold higher (*p* < 0.05) than in HPF, respectively.

Levels of PGE_2_ did not differ among healthy, early atretic-like follicles and IPC cysts but remained three to fourfold higher in SOC than 19-20PF, HPF, APF and IPC follicular/cystic fluid (*p* < 0.05; Fig. 1E). Concentration of PGFM was about threefold higher in HPF, APF and SOC than in IPC fluid (*p* < 0.05; Fig. 1F).

P_4_ concentration in follicular and cystic fluid was negatively correlated with E_2_ (r = − 0.5484, *p* < 0.0001), T (r = − 0.4820, *p* < 0.05) and A_4_ (r = − 0.6249, *p* < 0001). The ratio of P_4_ to E_2_, A_4_ and T in follicular and cystic fluid significantly increased in both cysts groups IPC and SOC when compared with the follicular phase follicles (Supplementary Fig. S4).

### Factors related to progesterone and estrogen synthesis

**Figure 2:**
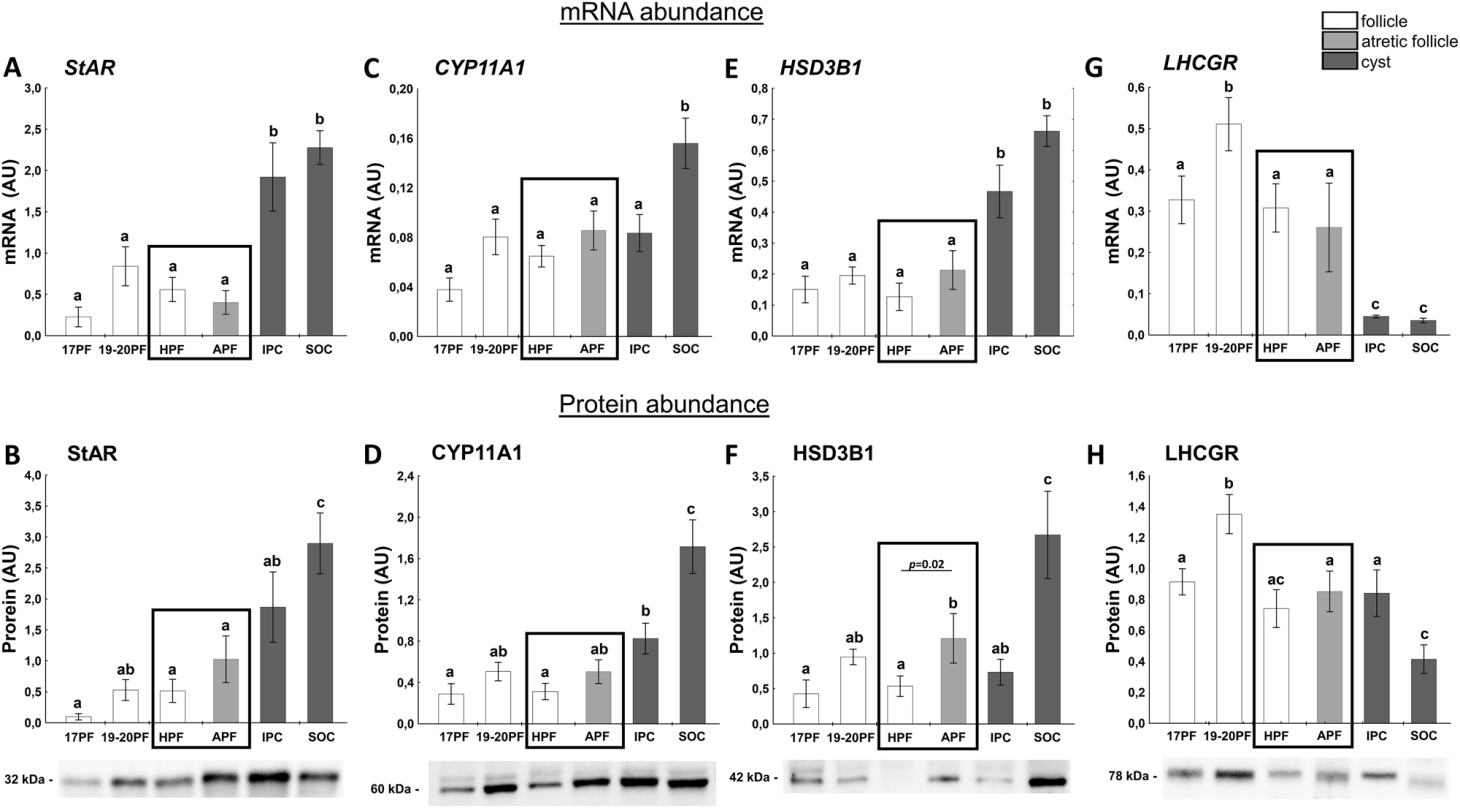
The expression of StAR (**A**,**B**), CYP11A1 (**C**,**D**), HSD3B1 (**E**,**F**), and LHCGR (**G**,**H**) in follicular (17PF, 19-20PF, HPF, APF) and cystic (IPC, SOC). Gene expression was normalized to the geometric mean of ACTB and GAPDH (AU), identified as the best reference genes by NormFinder algorithm. Protein levels were normalized to total protein content (AU) using TGX Stain-Free gel technology (**B**,**D**,**F**,**H**). Uncropped blots are presented in Supplementary Fig. S5 online. Data are presented as mean ± SEM. Data were analyzed using one-way ANOVA and LSD test by selecting appropriate planned comparisons. Letters (a, b) indicate a significant difference between groups (*p* < 0.05). For framed groups (follicles derived from ovaries of the same gilts), Student’s t-test was performed. AU – arbitrary units.

StAR, CYP11A1 and HSD3B1 are key factors involved in synthesis of P_4_^35^. The highest abundance of *StAR* and *HSD3B1* mRNA was found in cystic walls of IPC and SOC (*p* < 0.05) in comparison with follicular walls (Fig. 2A,E). Also the abundance of *CYP11A1* mRNA was highest (*p* < 0.05) in *SOC* (Fig. 2C). Expression of StAR and CYP11A1 protein was low in all classes of preovulatory follicles then increased in SOC (Fig. 2B,D). The significant increase of HSD3B1 protein expression in APF vs. HPF (Fig. 2F) could be the origin of increased P_4_ levels in APF follicles (Fig. 1D). The highest protein expression of StAR, HSD3B1 and CYP11A1 were found in the spontaneous cysts (Fig. 2B,D,F).

LHCGR signaling is required for steroidogenesis^36^. The highest level of *LHCGR* mRNA/protein abundance was obtained in 19-20PF. The slight but significant drop (*p* < 0.05) occurred in both preovulatory follicles—HPF and APF. Whereas *LHCGR* mRNA abundance was very low in both types of cysts (Fig. 2G), the protein levels in IPC was higher than SOC cyst (Fig. 2H; *p* < 0.05).

**Figure 3:**
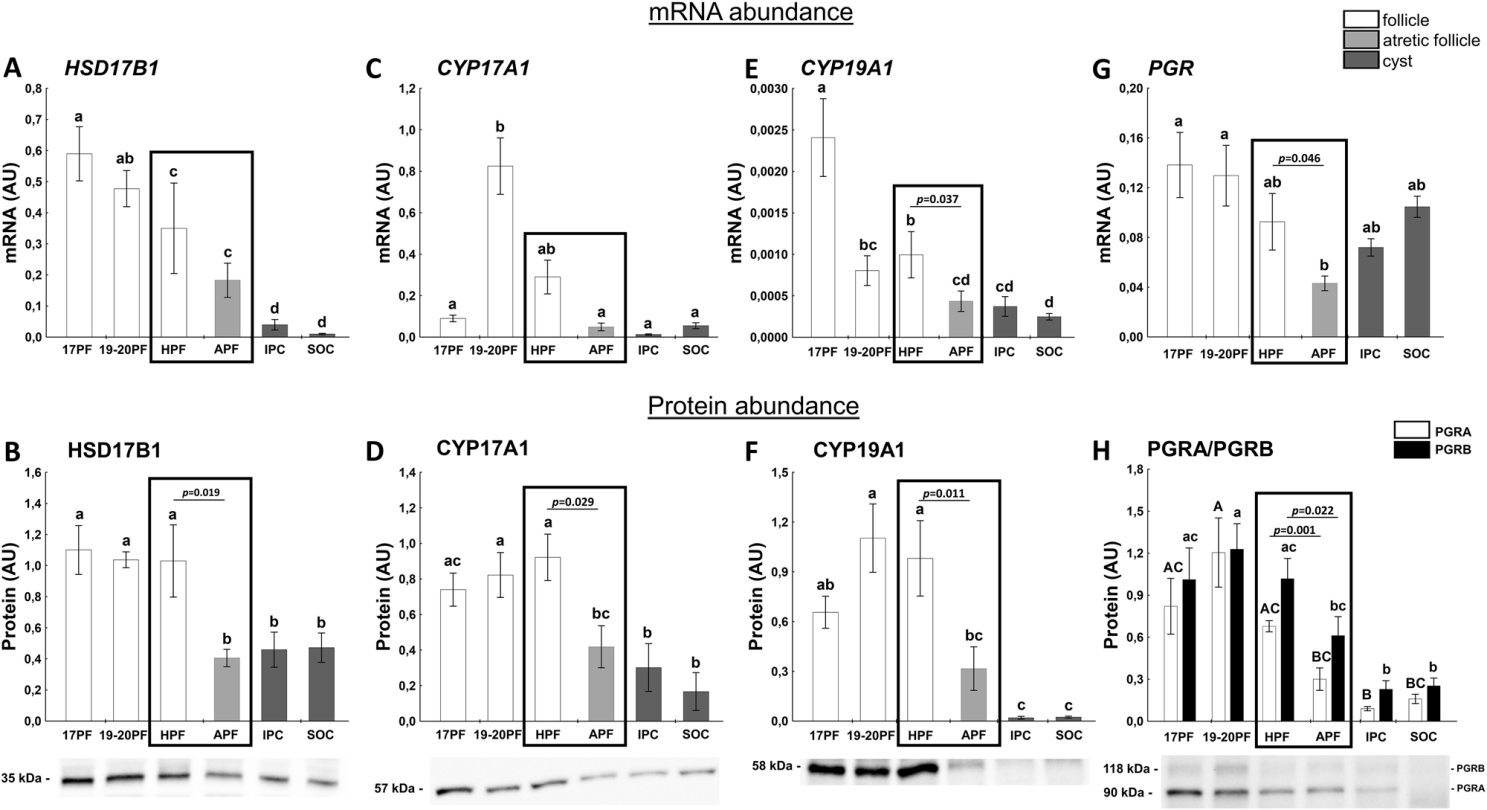
The expression of HSD17B1 (**A**,**B**), CYP17A1 (**C**,**D**), CYP19A1 (**E**,**F**), and PGR (**G**,**H**) in follicular (17PF, 19-20PF, HPF, APF) and cystic (IPC, SOC). Gene expression was normalized to the geometric mean of ACTB and GAPDH (AU), identified as the best reference genes by NormFinder algorithm. Protein levels were normalized to total protein content (AU) using TGX Stain-Free gel technology (**B**,**D**,**F**,**H**). Uncropped blots are presented in Supplementary Fig. S6 online. Data are presented as mean ± SEM. Data were analyzed using one-way ANOVA and LSD test by selecting appropriate planned comparisons. Letters (a, b) indicate a significant difference between groups (*p* < 0.05). For framed groups (follicles derived from ovaries of the same gilts), Student’s t-test was performed. *AU* arbitrary units.

Expression of *CYP17A1*, *HSD17B1* and *CYP19A1* mRNA, enzymes responsible for androgens and estrogens synthesis varied and was decreased in atretic-like follicles (Fig. 3A,C,E), when compared to 19-20PF. *HSD17B1* and *CYP19A1* mRNA remained low in induced or spontaneously occurred cysts (Fig. 3A,E; *p* < 0.05 vs. HPF and 19-20 PF). The protein abundance of these three enzymes was maintained high in 17PF, 19-20PF and HPF groups and the significant two to threefold drop occurred in APF and then remained lower in both types of cysts (Fig. 3B,D,F).

Expression of *PGR* mRNA was maintained relatively high in 17PF and 19-20PF groups, and the lowest levels were noticed for APF (*p* < 0.05). A t-test also showed lower *PGR* mRNA in APF vs. HPF (*p* = 0.046; Fig. 3G). Abundance of *PGR* mRNA in postovulatory cysts was comparable to the level occurred in the preovulatory follicles. Abundance of PGR proteins (protein A and B) followed the pattern of mRNA expression, dropped to the lowest level in the atretic-like preovulatory follicles (Fig. 3H), but contrary to mRNA expression, remained low in cysts.

StAR protein levels was positively correlated with CYP11A1 protein (r = 0.8594, *p* = 0.028), fluid P_4_ concentration (r = 0.6551, *p* < 0.0001) and negatively with CYP19A1 (r = − 0.3861, *p* = 0.03) and MMP2 (r = − 0.9251, *p* = 0.008) proteins. There was also a positive correlation between abundance of CYP19A1 protein and CYP17A1 protein (r = 0.3631, *p* = 0.032) and E_2_ concentration (r = 0.6013, *p* = 0.0001) and negative with P_4_ concentration (r = 0.5757; *p* < 0.01).

### Factors related to the production and action of prostaglandins (PGs)

**Figure 4:**
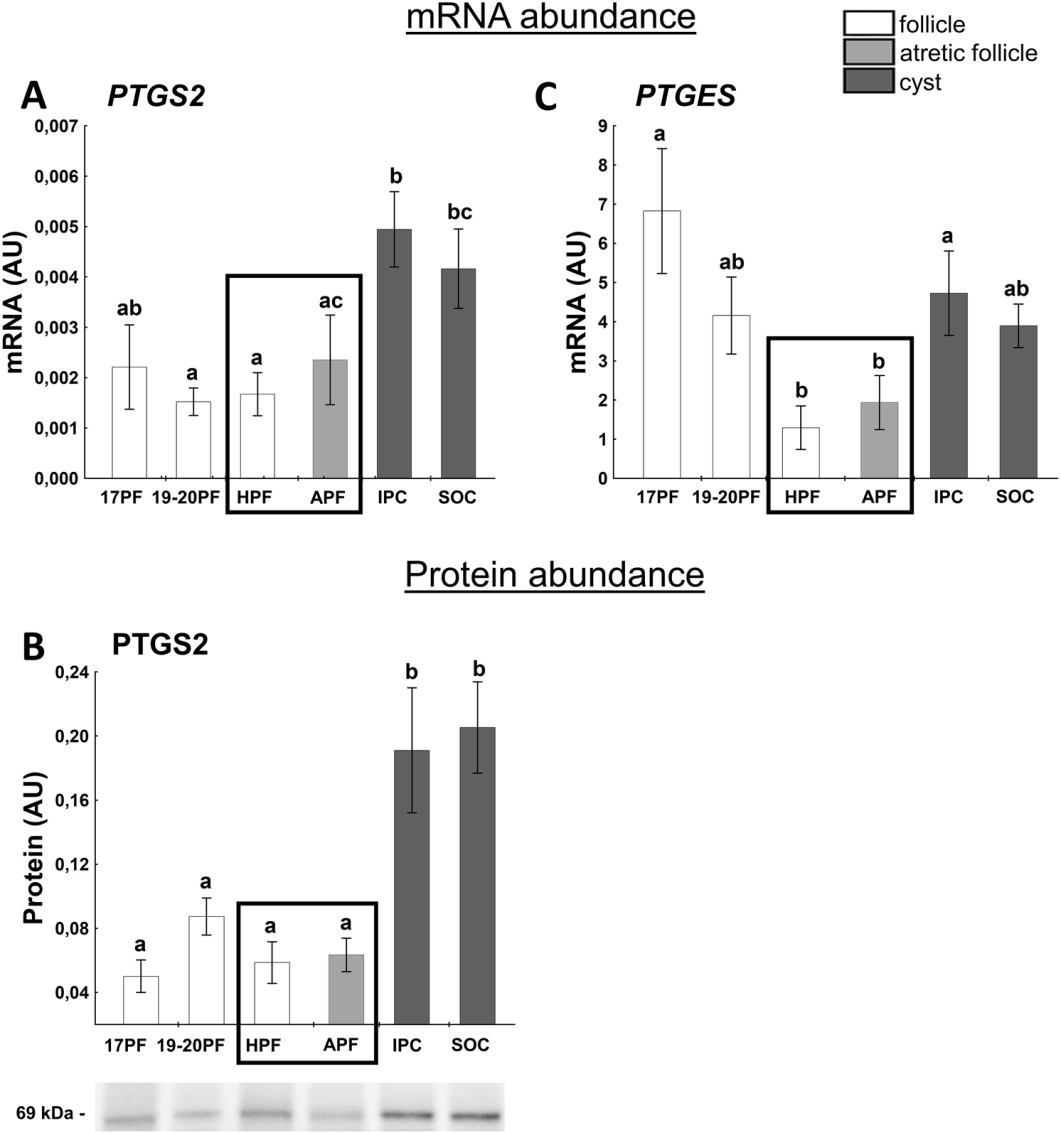
The expression of PTGS2 (**A**,**B**), PTGES (**C**) in follicular (17PF, 19-20PF, HPF, APF) and cystic (IPC, SOC) walls. Gene expression was normalized to the geometric mean of ACTB and GAPDH (AU), identified as the best reference genes by NormFinder algorithm. Protein levels were normalized to total protein content (AU) using TGX Stain-Free gel technology (**B**). Uncropped blots are presented in Supplementary Fig. S7 online. Data are presented as mean ± SEM. Data were analyzed using one-way ANOVA and LSD test by selecting appropriate planned comparisons. Letters (a, b) indicate a significant difference between groups (*p* < 0.05). For framed groups (follicles derived from ovaries of the same gilts), Student’s t-test was performed. *AU* arbitrary units.

The first rate-limiting step in the conversion of arachidonic acid to prostaglandin H2 (PGH_2_) is catalyzed by PTGS2 (COX-2), in the next step PGH_2_ is converted to PGE_2_, PGF_2a_ or prostacyclin and thromboxane. Both prostaglandins play important role in the development of preovulatory follicles and ovulation^37–39^. The higher abundance of *PTGS2* mRNA and protein (*p* < 0.05) was indicated in IPC and IPC/SOC cyst, respectively, compared with all preovulatory follicles (Fig. 4A,B). PTGS2 protein abundance was about threefold higher (*p* < 0.05) in IPC than HPF and APF (Fig. 4B). We found lower (*p* < 0.05) mRNA level of *PTGES* in HPF and APF than IPC and 17PF (about 4.4- and 2-fold, respectively; Fig. 4C).

### Factors related to MMP-TIMP system

The MMP-TIMP system is important for follicular development and rupture of the follicle wall during ovulation. Matrix metalloproteinase (MMP1 and MMP2) are collagenases, and their amounts increase in preovulatory follicle^40^, however tissue-specific inhibitors (TIMPs; i.e. TIMP-1 and TIMP-2) inhibit their activity, in consequence limiting follicular wall destruction^41^.

**Figure 5:**
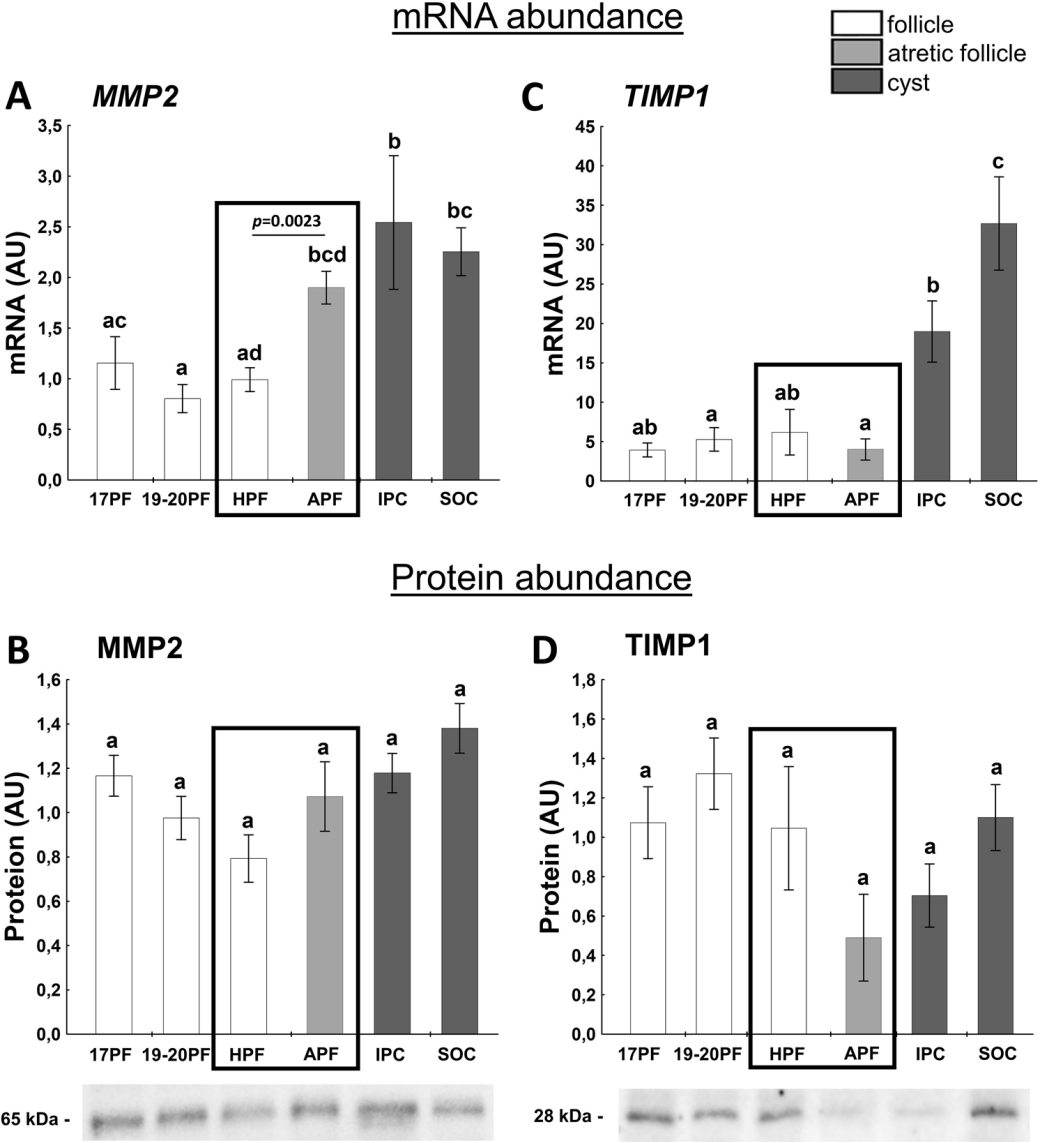
The expression of MMP2 (**A**,**B**), TIMP1 (**C**,**D**) in follicular (17PF, 19-20PF, HPF, APF) and cystic (IPC, SOC) walls. Gene expression was normalized to the geometric mean of ACTB and GAPDH (AU), identified as the best reference genes by NormFinder algorithm. Protein levels were normalized to total protein content (AU) using TGX Stain-Free gel technology (**B**). Uncropped blots are presented in Supplementary Fig. S7 online. Data are presented as mean ± SEM. Data were analyzed using one-way ANOVA and LSD test by selecting appropriate planned comparisons. Letters (a, b) indicate a significant difference between groups (*p* < 0.05). For framed groups (follicles derived from ovaries of the same gilts), Student’s t-test was performed. *AU* arbitrary units.

*MMP2* mRNA levels were higher in atretic-like preovulatory follicles and both cysts groups than in the preovulatory healthy follicles (Fig. 5A). *MMP2* mRNA abundance was 2.5- and 1.9-fold higher in APF (*p* = 0.0023) and IPC than in HPF.

Abundance of *TIMP1* mRNA remained constant in preovulatory follicles and then sharply increased in both cyst groups. Interestingly, IPC abundance was about fivefold higher than APF (Fig. 5C). However, we did not observe changes in expression of MMP2 and TIMP1 protein in follicular and cyst walls (Fig. 5B,D). There was a negative correlation between TIMP1 protein abundance and StAR protein (r = − 0.9251, *p* = 0.008) and the level of P_4_ (r = − 0.8838, *p* = 0.019) and E_2_ (r = − 0.8152, *p* = 0.048) in fluid.

### Factors related to local regulation of cell function

**Figure 6:**
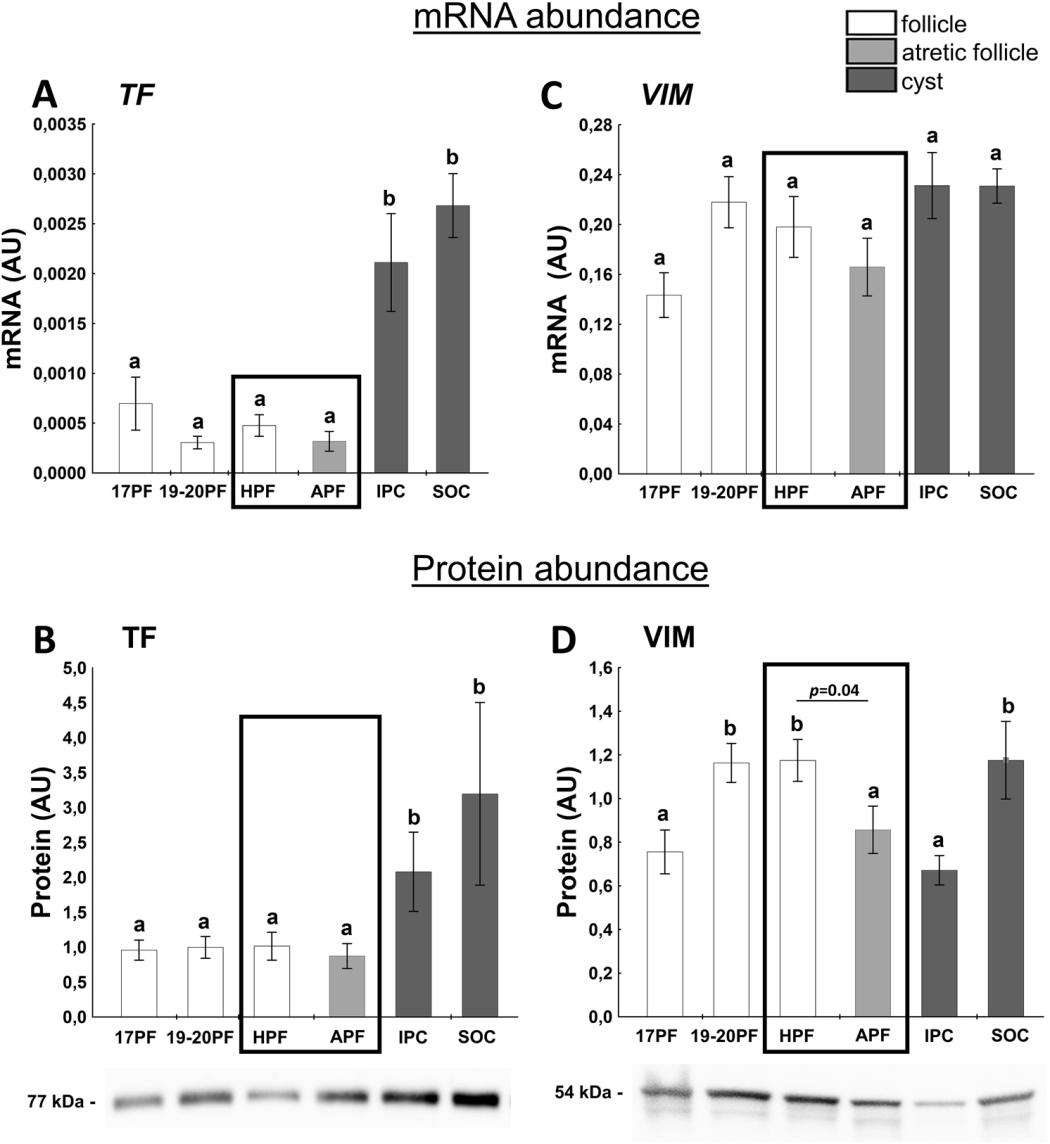
The expression of TF (**A**,**B**), VIM (**C**,**D**) in follicular (17PF, 19-20PF, HPF, APF) and cystic (IPC, SOC) walls. Gene expression was normalized to the geometric mean of ACTB and GAPDH (AU), identified as the best reference genes by NormFinder algorithm. Protein levels were normalized to total protein content (AU) using TGX Stain-Free gel technology (**B**). Uncropped blots are presented in Supplementary Fig. S8 online. Data are presented as mean ± SEM. Data were analyzed using one-way ANOVA and LSD test by selecting appropriate planned comparisons. Letters (a, b) indicate a significant difference between groups (*p* < 0.05). For framed groups (follicles derived from ovaries of the same gilts), Student’s t-test was performed. *AU* arbitrary units.

Transferrin (TF) plays a crucial role in the local regulation of ovarian function^42^. Abundance of *TF* mRNA/protein were higher in cysts (IPC and SOC) than in all follicular samples (Fig. 6A). Interestingly, TF protein was about two to threefold higher in IPC compared with HPF and APF (*p* < 0.05; respectively; Fig. 6B).

Vimentin (VIM) is involved in organelle transport, cell migration and proliferation^43^. We did not observe changes in *VIM* mRNA expression in experimental groups (Fig. 6C). However, VIM protein levels were higher in 19-20PF, HPF, and SOC than in 17PF, APF and IPC (Fig. 6D, p < 0.05).

### miRNA in follicular and cyst walls

**Figure 7:**
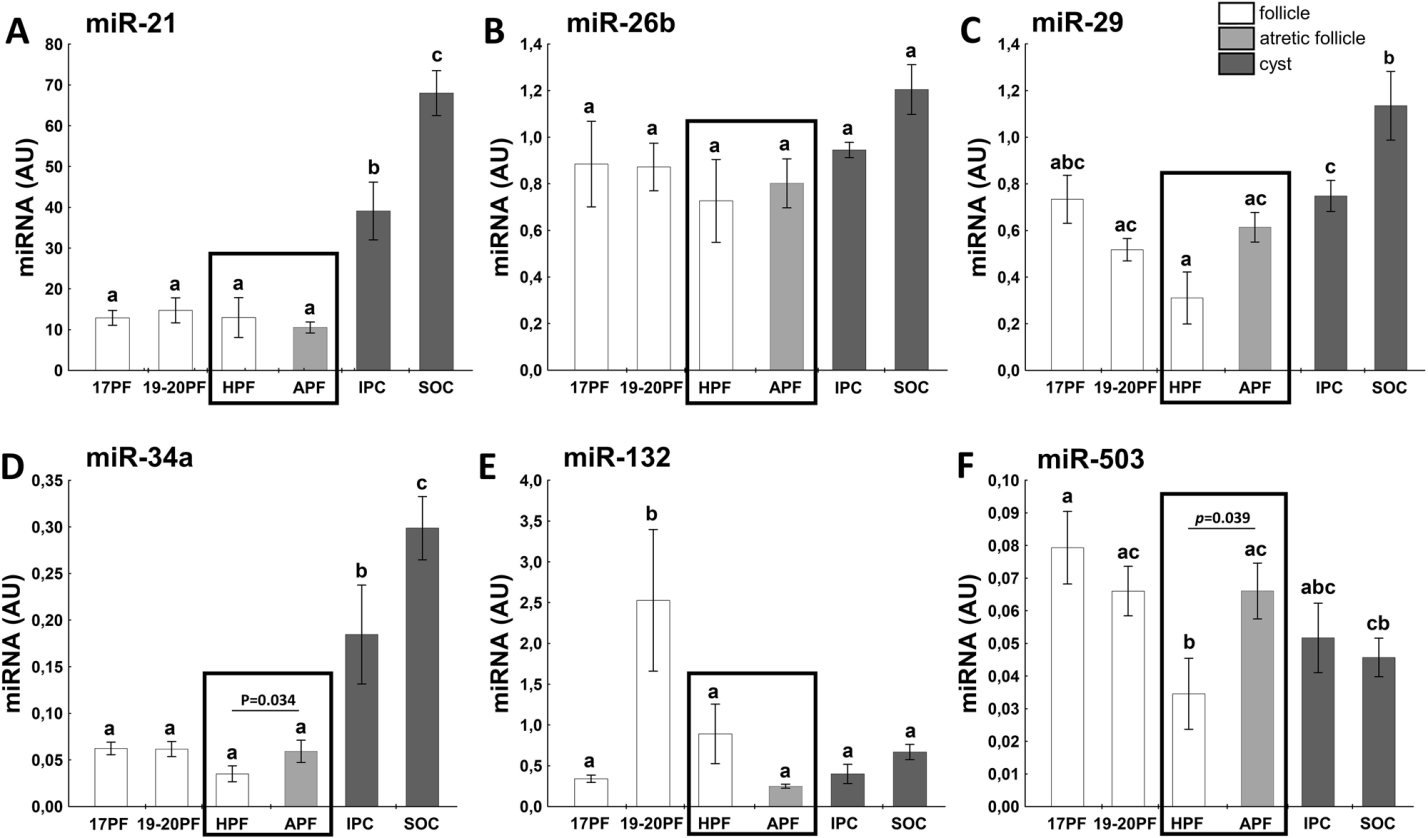
The expression of miR-21 (**A**), miR-26b (**B**), miR-29 (**C**), miR-34a (**D**), miR-132 (**E**), and miR-503 (**F**) in follicular (17PF, 19-20PF, HPF, APF) and cystic (IPC, SOC) walls. Data are presented as mean ± SEM. Data were analyzed using one-way ANOVA and LSD test by selecting appropriate planned comparisons. Letters (a, b) indicate a significant difference between groups (*p* < 0.05). For framed groups (follicles derived from ovaries of the same gilts), Student’s t-test was performed. *AU* arbitrary units.

Based on our earlier results (data no shown), we selected six miRNAs involved in follicular growth and steroidogenesis pathway. The expression of miR-21 and miR-34a in walls of IPC and SOC was much higher (Fig. 7A,D) than in all follicular samples. There was about 3- and fourfold higher expression of miR-21 and about 6- and threefold higher expression of miR-34 in IPC compared with HPF and APF, respectively. Interestingly, expression of miR-34a was significantly higher in APF than HPF (*p* = 0.034, Fig. 7D). There were correlations between miR-21 and miR-29 (r = 0.6442, *p* = 0.0001) and miR34a (r = 0.7529, *p* = 0.0001) and between miR-34a and miR-29 (r = 0.5059, *p* = 0.001). Moreover, abundance of both miR-21 and miR-34a correlated with E_2_ and P_4_ in follicular/cystic fluid.

We did not observe changes in expression of miR-26b in follicular and cyst walls (Fig. 7B), however abundance of miR-26b correlated with miR-29 (r = 0.6087, *p* = 0.0001). Similar almost constant expression was noticed for miR-132 (Fig. 7E), except 19-20PF where the highest abundance was observed when compared with other group (*p* < 0.05).

Abundance of miR-29 was about 2.5-fold higher in IPC than HPF (*p* < 0.05), and was the highest in SOC group (Fig. 7C). In addition, expression of miR-29 positively correlated with concentration of P_4_ (r = 0.3894, *p* = 0.012) in follicular/cystic fluid.

Expression of miR-503 gradually decreased in preovulatory follicles (17PF, 19-20PF), reaching the lower level in healthy follicles (*p* < 0.05, Fig. 7F), but the twofold rebound of its abundance occurred in counterparts atretic follicles (*p* = 0.039, Fig. 7F).

### TNFα mRNA expression in follicular/cystic walls and content in follicular/cystic fluid

**Figure 8:**
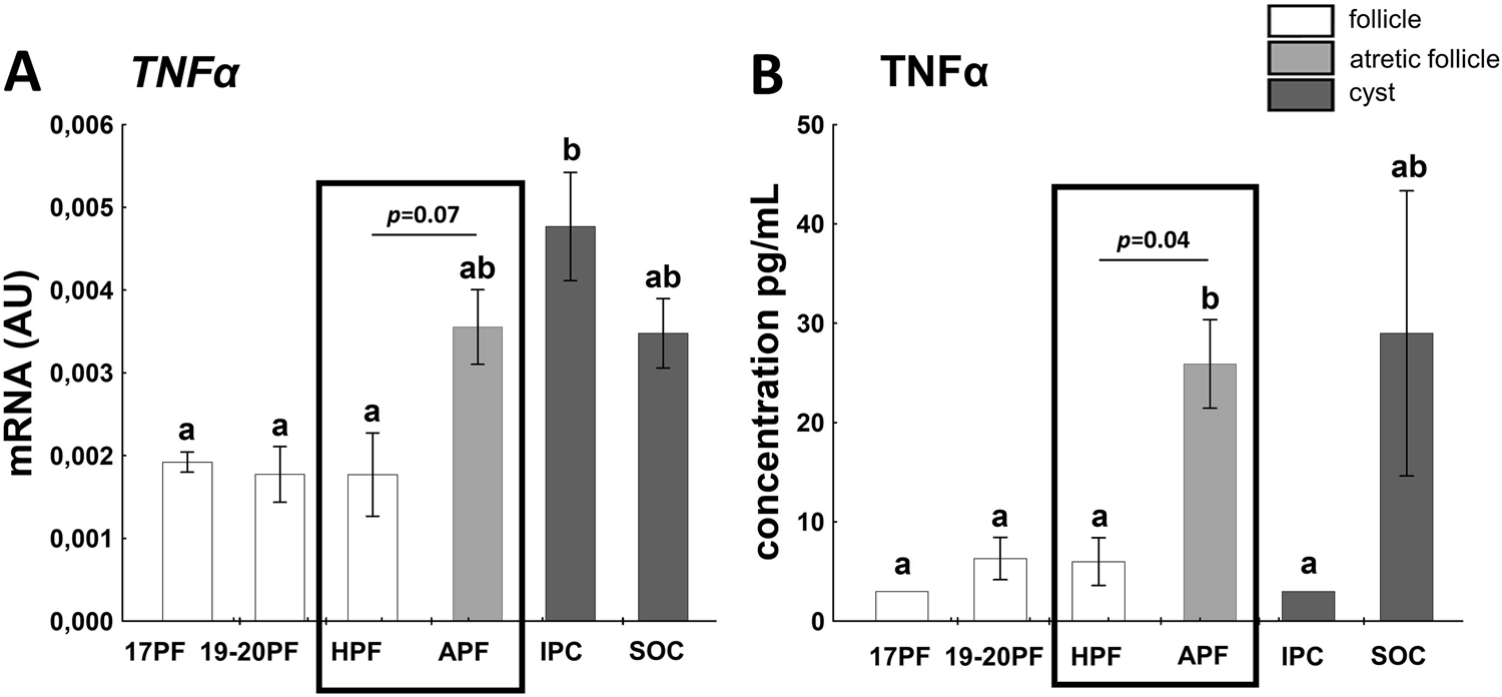
The expression of TNFα mRNA (**A**) and concentration of TNFα (**B**) in follicular (17PF, 19-20PF, HPF, APF) and cystic (IPC, SOC) walls/fluid. Gene expression was normalized to the geometric mean of ACTB and GAPDH (AU), identified as the best reference genes by NormFinder algorithm. Data are presented as mean ± SEM. Data were analyzed using one-way ANOVA and LSD test by selecting appropriate planned comparisons. Letters (a, b) indicate a significant difference between groups (*p* < 0.05). For framed groups (follicles derived from ovaries of the same gilts), Student’s t-test was performed. *AU* arbitrary units.

A cytokine TNFα induces several responses in cell, is involved in apoptosis and necrotic cell death^44^ and/or may serve as an immune cell marker. The TNFα levels remained constant in three classes of preovulatory follicles (17PF, 19-20PF and HPF) and increased in atretic follicles (*p* = 0.07) and postovulatory induced cysts (*p* < 0.05, Fig. 8A). The similar concentration of TNFα remained in 17PF, 19-20PF and HPF, but increased 4.5-fold in APF follicular fluid (25.89 vs. 5.79 pg/mL, *p* < 0.05, Fig. 8B). Interestingly, the TNFα level was maintained low in hormonally induced cyst (vs. APF, *p* < 0.05), while high intergroup variation occurred in spontaneously occurred cysts.

## Discussion

The present study provides a detailed description of the hormonal profile of healthy and atretic follicles, as well as ovarian cysts having different developmental background, i.e., those which spontaneously occur in herds or hormonally induced by gonadotropins. Profiling of factors involved in progesterone and estrogen synthesis, the production and action of PGs, the local regulation of cell function, and the MMP-TIMP system provides comprehensive data on the molecular environment in healthy and oligocystic porcine ovaries. Additional profiling of miRNAs, potent gene expression regulators known for their action in ovarian pathophysiology, hinted at the possible involvement of non-coding RNAs in the regulation of ovarian cyst formation in pigs.

A comparison of steroid hormone concentration in follicular fluid of intact gilts (19-20PϜ) and in healthy, hormonally induced preovulatory follicles, found no difference in concentrations of E_2,_ P_4_ and PGE_2_. On the other hand, the follicular fluid of atretic-like preovulatory follicles showed high concentration of P_4_ and much lower levels of E_2_ and A_4_ compared to healthy PFs. Concentrations of E_2_, A_4_, T and PGE_2_ in those follicles were comparable to concentrations found in the cystic fluid of experimentally and spontaneously occurred ovarian cysts. However, the level of P_4_ remained relatively high in atretic follicles, and was doubled after ovulation in hormonally induced and spontaneously occurring cysts. This observation supports our hypothesis that early atretic-like preovulatory follicles are transformed into cysts. The higher concentration of P_4_ in spontaneously occurring than hormonally induced cysts can be explained by differences in collection time. Ovaries from SOC gilts were collected during the mid-luteal phase of the estrous cycle, while gonadotropin-induced ovaries were collected a few days after ovulation. A similar explanation can be given for the much higher levels of prostaglandins in SOC gilts. Previous studies showed comparable concentrations of estrogen, androgens and progesterone in cysts of oligocystic ovaries in sows^4^ and gonadotropins induced postovulatory cyst in our experiment. However, the concentration of E_2_ in the cystic fluid of polycystic ovaries was approximately 100-fold higher than in oligocystic ovaries. Cysts from polycystic ovaries produced significantly larger quantities of E_2_, T and A_4_ than cyst of oligocystic ovaries, while maintaining similar concentrations of P_4_^4^. The hormonally induced and spontaneously occurred cysts in our study did not differ much from cysts occurring in oligocystic ovaries. It is very likely that etiopathogenesis of cysts in oligo- and polycystic ovaries differs, which awaits further studies.

Our studies point at important aspects of early designation of follicles towards cysts formation. Comparison of mutual ratios of steroid hormones in the atretic-like preovulatory follicles and cysts clearly showed earlier gestagenization, but not androgenization, of the preovulatory follicles stigmatized to become cysts after ovulation. In women, an excess of androgen, and elevated expression of LH/hCG receptor and CYP17A1 are hallmarks of polycystic ovary syndrome (PCOS)^45^. In contrast, the concentration of androgens and expression of CYP17A1 protein remained low in porcine lutein cysts. Furthermore, the main difference between the course of folliculogenesis during PCOS in women and cyst development in pigs concerns all classes of primary, growing and maturing follicles, which appear in considerably higher numbers in women then in the pig^4^.

The increased production of P_4_ was associated with elevation of StAR, CYP11A1 and HSD3B1 protein abundance – enzymes directly involved in P_4_ synthesis. A reduction in the protein expression of CYP17A1 (the enzyme transforming pregnenolone to androstenedione) was accompanied by decreased levels of CYP19A1 and HSD17B1, both involved in the final synthesis of E_2_. Our study confirmed the earlier reports^13^ that neither *LHCGR* mRNA/protein expression nor secretion of PGE_2_ and PGF_2α_ differ between follicles designed to form cysts and follicles assigned to form CL. It is also consistent with a hypothesis that formation of cysts in response to exogenous gonadotropin stimulus is not due to the insensitivity of follicles to LH/hCG (at least on the level of gonadotropins receptor) but depends on the prevailing steroid hormone milieu. An interruption of ovulatory cascade, presumably by eCG and/or hCG, leading to earlier luteinization of follicular walls may prevent rupture of the atretic preovulatory follicles, which become cysts.

This is in contrast to estrogenic, healthy follicles, which ovulate at designated time of the estrous cycle. Interestingly, eCG-stimulated preovulatory follicles seem to be more susceptible to atresia than are follicles after FSH stimulation^27^. In the atretic preovulatory follicles, the ovulatory triggers, exogenous hCG, or the native LH preovulatory surge, enhance the luteinization of follicular walls, as reflected in the high P_4_/E_2_ ratio observed in gonadotropin-induced and spontaneously occurring cysts.

The progesterone receptor (PGR)—a steroid receptor transcription factor—is activated by high local concentration of P_4_ and translocated to the nucleus, where transcription of downstream targets crucial for follicular rupture is initiated^46^. Knockdown of PGR blocks ovulation in mice^47^ and rhesus monkey^48^. Interestingly, we showed a parallel drop in *PGR* mRNA and protein abundance in early atretic-like preovulatory follicles, which are precursors of ovarian cysts. Whereas the mRNA is rebounded in the cysts’ walls, both PGR-A and PGR-B protein expression remained low in the developed cysts. Recently, Robker et al.^47^ found that two proteins involved in ovulation processes, ADAMTS-1 and cathepsin, are transcriptional targets of PGR and play a crucial role in follicular rupture. The low expression of PGR in early atretic preovulatory follicles could be an important step in postponing their rupture after an endogenous LH surge onset or hCG administration.

Since the cellular and molecular mechanism of the preovulatory follicle differentiation and ovulation is very complexed^49^ we tried to estimate the role of local regulators, i.e. TF and VIM, in follicle development and potential transformation into cyst. Recently we showed localization of TF and VIM in the porcine ovarian follicle^24^. However, spatial and temporal changes in the expression of TF and VIM in preovulatory follicles and postovulatory cysts are not satisfactory to attribute them a significant role in the transformation of atretic preovulatory follicles into cysts.

It is known that miRNAs, a class of small, non-coding RNA, which regulate the expression of many genes involved in processes governing follicular development^50^, including atresia and ovulation. A different expression of miRNA between dominant and subordinate or dominant versus luteinized follicles in sheep suggest the involvement of miRNA in follicle selection process and ovulation^51^. The most intriguing difference in miRNA profiles of preovulatory healthy and atretic follicles occurred for miR-503. The decrease of miR-503 abundance in the ovarian follicular fluid concurs with previously observed response to eCG treatment in mice that were given a follow-up, ovulatory dose of hCG^52^. The different expression of this miRNA during the peri-ovulatory period in mice^52^ and sheep^53^ indicated its involvement in governing ovarian function. Furthermore, the ovary is also the main site of miR-503 expression in human^54^. In sheep miR-503 levels also transiently decreases in preovulatory follicles before rebounding in corpora lutea^53^. It remains to be confirmed whether the gradual drop in miR-503 transcript (similar to the observed decline in miR-29) in the healthy follicle triggers the ovulatory cascade. It is worth noting the high abundance of miR-503 in the counterpart atretic-like preovulatory follicles and its correlation with elevated P_4_ and StAR/HSD3B1 protein levels. Thus it seems likely that miR-503, and perhaps miR-29, are acting together in transformation of the preovulatory atretic follicles into lutein cysts, but it is without doubt a promising pattern for further research.

Follicle atresia is mainly described in the preantral and small antral follicles^55^. The elements of follicle atresia mechanism are well developed in early antral follicles^56^. The preovulatory follicles rarely undergo atresia^57^. Treatment with TNFα induced apoptotic changes in granulosa cells^56^ and stimulated progesterone production in rodent healthy, atretic^58,59^, and preovulatory^60^ follicles. TNFα inhibits FSH-stimulated aromatase activity in granulosa cells^61^. In general, an elevation of progesterone and a decrease of estradiol is observed in follicles undergoing atresia^46,47^. Although the classification of preovulatory atretic-like follicles was made based on the morphological observations during their surgical collection, the high concentration of TNFα and other endocrine parameters proves that those follicles were atretic. TNFα could be important factor indicating that the atretic follicles will soon undergo apoptosis. In general, TNFα mRNA levels in walls of follicles and cysts were parallel to TNFα protein concentration in follicular/cystic fluid in the status dependent manner—low in all classes of healthy preovulatory and high in the atretic follicles and cysts. In contrast, for hormonally induced cysts TNFα protein and secretion was dramatically decreased, suggesting posttranscriptional regulation.

In conclusion, the exogenous gonadotropin (eCG, hCG)-induced and the spontaneously occurred ovarian cysts in oligocystic ovaries of gilts are characterized by similarities in their steroid hormone profiles and molecular regulation. Both types of cysts were classified here as follicular lutein cysts. The lutein cysts differ markedly from healthy preovulatory follicles in terms of the lower concentration of estrogens and androgens, and higher level of P_4_. These features of lutein cysts are caused by the lower expression of HSD17B1, CYP17A1 and CYP19A1 and/or the higher abundance of StAR and HSD3B1 proteins, respectively. The atretic preovulatory follicles before ovulation are also distinguished by a low expression of PGR, high level of TNFα and miR-503 in comparison with ready-to-ovulate, heathy, preovulatory follicles. We suggest that cysts could be recruited from the early atretic preovulatory follicles that irretrievably lost their estrogenic milieu, with a shift from estradiol to progesterone synthesis. Such follicles are unable to ovulate due to the earlier luteinization and possible disruption of the ovulatory cascade. These results seem to change the present paradigm that the insignificant LH preovulatory surge is the reason for cyst occurrence in the pig. Furthermore, our presented data clearly shows that our method of ovarian cyst induction in prepubertal gilts can be useful in further studies on the etiology of lutein oligocyst formation in the pig and other species, including cattle and human.

## Supplementary Information

Supplementary Information.

## Data Availability

None of the data were deposited in an official repository. Data are available upon request from the corresponding authors.
