# Supplementary material for: Atretic preovulatory follicles could be precursors of ovarian lutein cysts in the pig
Source: Sci Rep. 2023 May 12;13:7758. doi: 10.1038/s41598-023-34563-4 (PMC10182091; doi:10.1038/s41598-023-34563-4)
Supplement: Supplementary file 1 — Supplementary Information. [file 41598_2023_34563_MOESM1_ESM.pdf]

Supplementary information

Atretic preovulatory follicles could be precursors of lutein ovarian cysts in the pig

Adam J. Ziecik, Pawel Likszo, Jan Klos, Katarzyna Gromadzka-Hliwa, Katarzyna Knapczyk-Stwora, Olli Peltoniemi, Zdzislaw Gajewski, Monika M. Kaczmarek

Supplementary Figures:

Supplementary Figure S1. Representative pictures showing ovaries classified.

Supplementary Figure S2. Representative images of hematoxylin and eosin stained spontaneously appeared follicular lutein cyst (A) and follicular lutein cysts.

Supplementary Figure S3. Example of antibodies specificity test.

Supplementary Figure S4. The mutual ratios for concentration of steroid hormones in follicular (17PF, 19-20PF, HPF, APF) and cystic (IPC, SOC) fluid.

Supplementary Figure S5. Uncropped blots for STAR, CYP11A1, HSD3B1 and LHCGR protein expression in preovulatory follicles and postovulatory cysts in gilts.

Supplementary Figure S6. Uncropped blots for HSD17B1, CYP17A1, CYP19A1 and PGRA/PGRB protein expression in preovulatory follicles and postovulatory cysts in gilts.

Supplementary Figure S7. Uncropped blots for PTGS2, MMP2 and TIMP1 protein expression in preovulatory follicles and postovulatory cysts in gilts.

Supplementary Figure S8. Uncropped blots for TF and VIM protein expression in preovulatory follicles and postovulatory cysts in gilts.

Supplementary Tables:

Supplementary Table S1. Number of visible ovarian follicles on both ovaries.

Supplementary Table S2. List of antibodies used in the study.

Supplementary Table S3. List of genes used in real-time PCR.

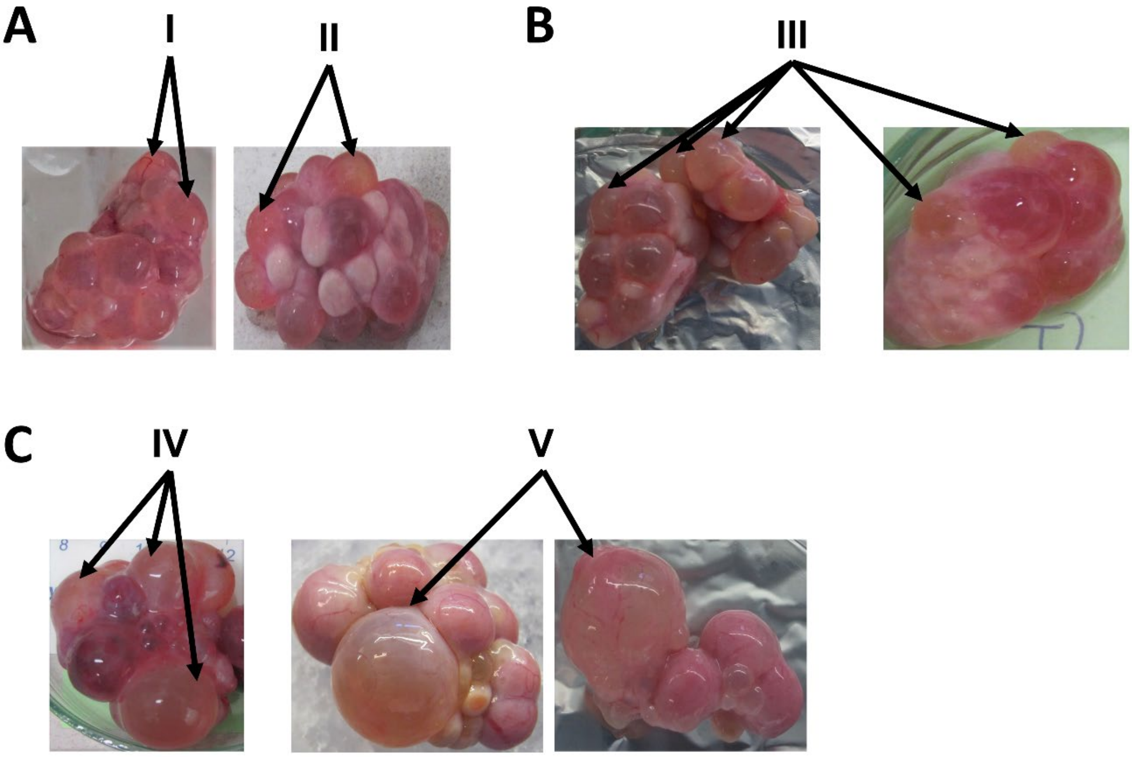

Supplementary Figure S1. Representative pictures showing ovaries classified as: A – gonadotropin-induced healthy preovulatory follicles (I) and gonadotropin-induced intact healthy preovulatory follicles (II); B – atretic-like preovulatory follicles (III); C – gonadotropin-induced provoked (IV) and spontaneously occurred (V) follicular cysts.

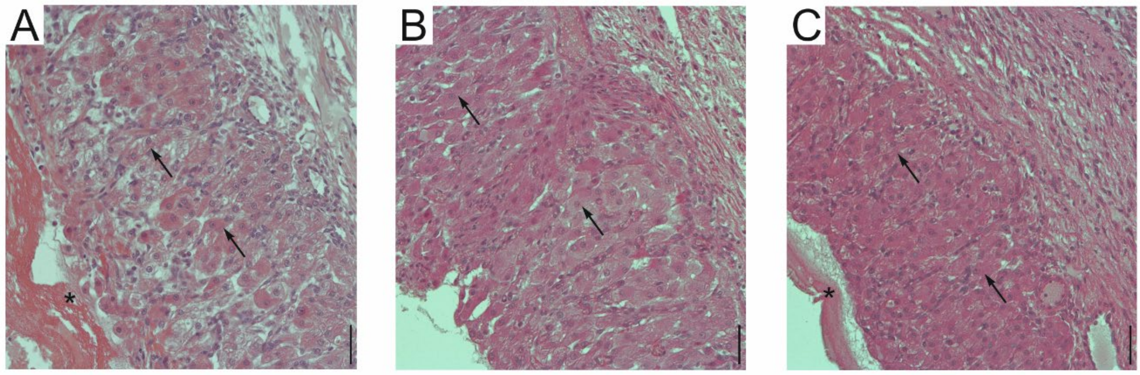

Supplementary Figure S2. Representative images of hematoxylin and eosin stained spontaneously appeared follicular lutein cyst (A) and follicular lutein cysts obtained from gilts after estrous cycle synchronization (B, C). Arrows indicate granulosa lutein cells; asterisks indicate connective tissue. Scale bars = 50 μm.

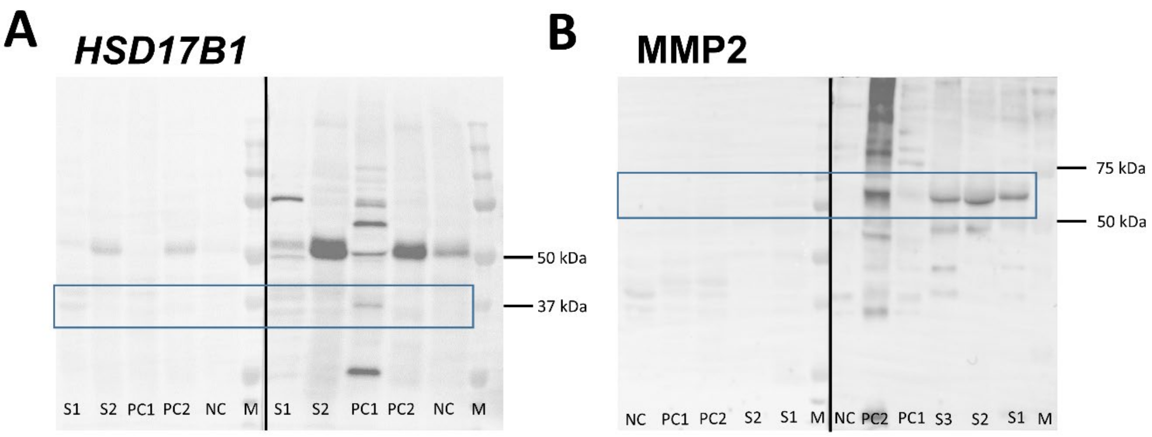

Supplementary Figure S3. Example of antibodies specificity test. Left panels represent membrane showing only secondary antibodies, whereas right panels represent membrane showing both primary and secondary antibodies. M, marker; S1 – S3, protein extracts from follicular or cystic walls; PC1, positive control (mouse brain); PC2, positive control (swine brain); NC, negative control (muscle). Blue boxes indicate expected position HSD17B1 or MMP2 protein, based on the predicted molecular weight.

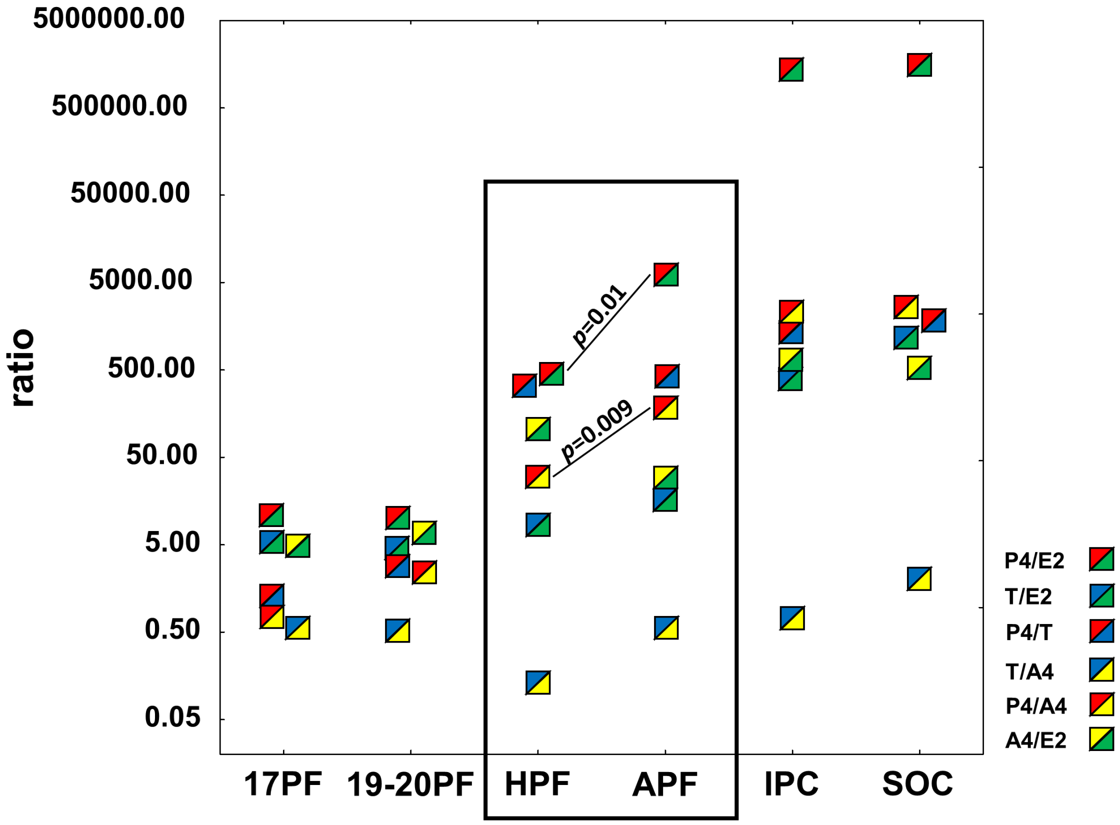

Supplementary Figure S4. The mutual ratios for concentration of steroid hormones in follicular (17PF, 19-20PF, HPF, APF) and cystic (IPC, SOC) fluid.

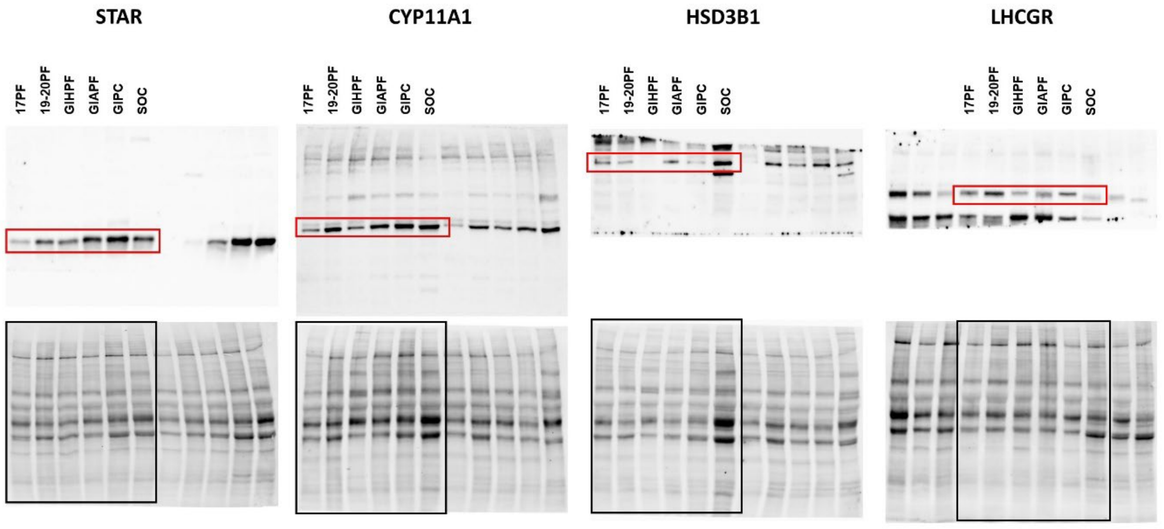

Supplementary Figure S5. Uncropped blots for STAR, CYP11A1, HSD3B1 and LHCGR protein expression in preovulatory follicles and postovulatory cysts in gilts. In each upper panel, full blot showing STAR, CYP11A1, HSD3B1 and LHCGR protein expression are showed. Lower panels represent equivalent TGX Stain-Free gel showing total protein. Red boxes indicate areas presented in Figure 2.

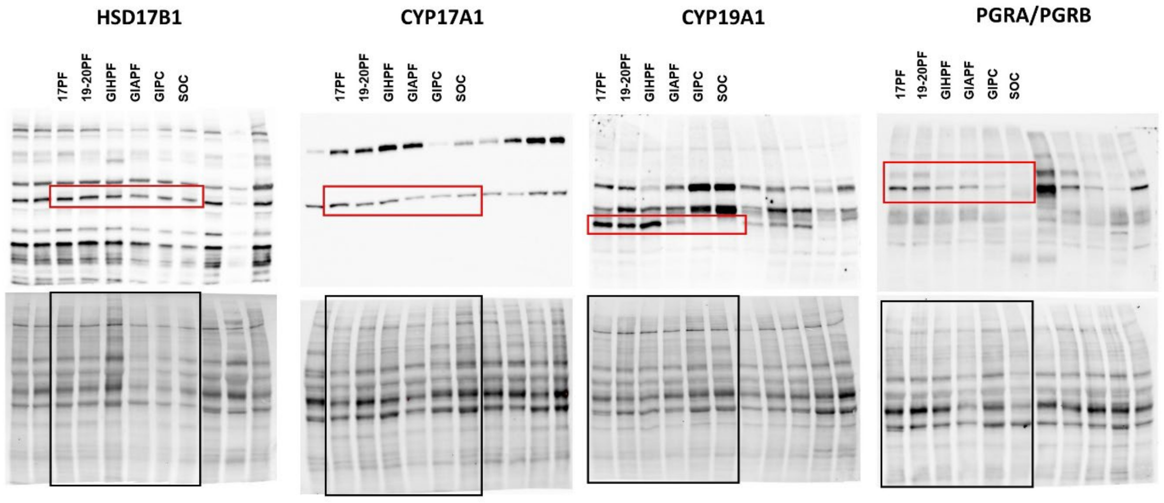

Supplementary Figure S6. Uncropped blots for HSD17B1, CYP17A1, CYP19A1 and PGRA/PGRB protein expression in preovulatory follicles and postovulatory cysts in gilts. In each upper panel, full blot showing HSD17B1, CYP17A1, CYP19A1 and PGRA/PGRB protein expression are showed. Lower panels represent equivalent TGX Stain-Free gel showing total protein. Red boxes indicate areas presented in Figure 3.

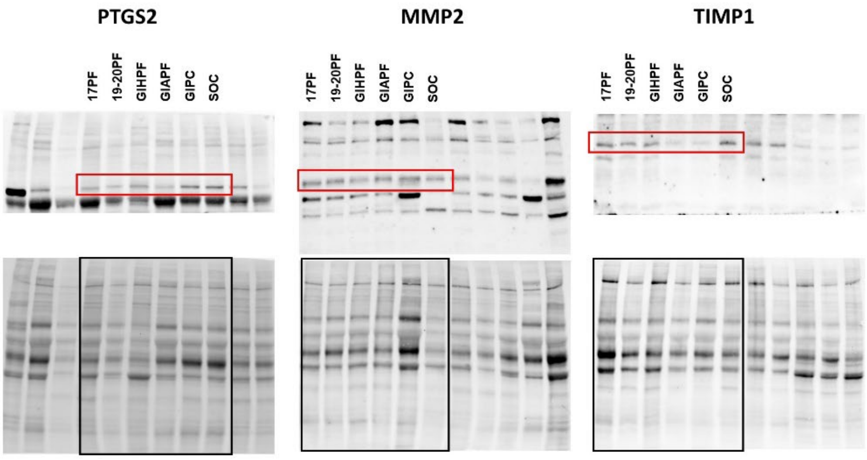

Supplementary Figure S7. Uncropped blots for PTGS2, MMP2 and TIMP1 protein expression in preovulatory follicles and postovulatory cysts in gilts. In each upper panel, full blot showing PTGS2, MMP2 and TIMP1 protein expression are showed. Lower panels represent equivalent TGX Stain-Free gel showing total protein. Red boxes indicate areas presented in Figure 4 and 5.

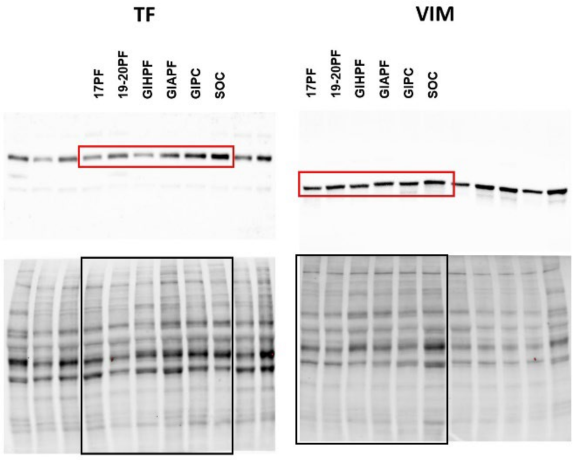

Supplementary Figure S8. Uncropped blots for TF and VIM protein expression in preovulatory follicles and postovulatory cysts in gilts. In each upper panel, full blot showing TF and VIM protein expression are showed. Lower panels represent equivalent TGX Stain-Free gel showing total protein. Red boxes indicate areas presented in Figure 6.

Supplementary Table S1. Number of visible ovarian follicles on both ovaries (healthy and early atretic) in different size categories; 30 h after hCG challenge in eCG-primed prepubertal gilts.

Follicles / Gilt identification	Healthy			Early atretic	Total
	<3 mm	3.1 - 5.9 mm	>6 mm	>6 mm	
J163	16	9	10	2	37
J164	3	3	17	2	25
J165	34	4	0	2	40
J187	5	11	10	3	29
J188	16	7	0	2	25
J192	6	9	2	2	19
J194	2	7	5	2	16
Mean	11.71	7.14	6.29	2.14	27.29

Supplementary Table S2. List of antibodies used in the study.

Peptide/Protein Target	Name of Antibody	Catalog No., Name of Source	Species Raised in Monoclonal or Polyclonal	Dilution used
**Prostaglandin assays**				
PGE_2	Anti-Prostaglandin E_2 Antibody	P5164 Sigma-Aldrich	Rabbit, polyclonal	1:200
PGFM	Anti-PGFM serum	WS4468-7 donated by Dr William Silvia	Rabbit, polyclonal	1:10000
**Western blot**				
CYP11A1	Anti- CYP11A1 Antibody	ab175408 Abam	Rabbit, polyclonal	2.2 µg/mL
CYP17A1	Anti- CYP17A1 Antibody	ab125022 Abcam	Rabbit, monoclonal	0.3 µg/mL
CYP19A1*	Anti-CYP19A1 Antibody	MCA2077S Bio-Rad	Mouse, monoclonal	1:250
HSD3B1	Anti- HSD3B1 Antibody	ab55268 Abcam	Mouse, monoclonal	1 µg/mL
HSD17B1	Anti- HSD17B1 Antibody	Sc26963 Santa Cruz Biotechnology	Goat, polyclonal	2 µg/mL
LHCGR	Anti- LHCGR Antibody	AD2716317 donated by Dr Marco Banoni	Mouse, monoclonal	1:200
PTGFS*	Anti- PTGFS Antibody	AV48180 Sigma	Rabbit, polyclonal	1 µg/mL
PTGS2	Anti-PTGS2 Antibody	160107 Cayman Chemical	Rabbit, polyclonal	5 µg/mL
StAR	Anti-StAR Antibody	ab96637 Abcam	Rabbit, polyclonal	2.5 µg/mL
PGRA/PGRB	Anti-PGRA Antibody	ab191138 Abcam	Rabbit, polyclonal	0.5 µg/mL
MMP2*	Anti-MMP2 Antibody	ab97779 Abcam	Rabbit, polyclonal	0.4 µg/mL
TIMP1	Anti- TIMP1 Antibody	sc21734 Santa Cruz Biotechnology	Mouse, monoclonal	0.5 µg/mL
TF	Anti-Transferrin Antibody	ab82411 Abcam	Rabbit, polyclonal	1 µg/mL
VIM	Anti-Vimentin Antibody	sc-6260 Santa Cruz Biotechnology	Mouse, monoclonal	0.2 µg/mL
Anti-rabbit, secondary antibodies	Immun-Star Goat Anti-Rabbit (GAR)-HRP Conjugate	1705046 Bio-Rad	Goat, polyclonal	1:20000
Anti-mouse, secondary antibodies	Immun-Star Goat Anti-Mouse (GAM)-HRP Conjugate	1705047 Bio-Rad	Goat, polyclonal	1:20000
Anti-goat, secondary antibodies	Goat IgG HRP-conjugated Antibody	HAF109 R&D Systems	Donkey, polyclonal	1:1000

* Predicted to interact with porcine antigen.

Supplementary Table S3. List of genes used in real-time PCR.

Genes symbol	Gene name	TaqMan Assays ID
CYP11A1	Cytochrome P450 Family 11 Subfamily A Member 1	Ss03384849_u1
CYP17A1	Cytochrome P450 Family 17 Subfamily A Member 1	Ss03394945_m1
CYP19A1	Cytochrome P450 Family 19 Subfamily A Member 1	Ss03384876_u1
HSD17B1	Hydroxysteroid 17-Beta Dehydrogenase 1	Ss04245960_g1
HSD3B1	Hydroxy-delta-5-steroid Dehydrogenase, 3 Beta- and Steroid Delta-isomerase 1	Ss03391752_m1
LHCGR	Luteinizing Hormone/Choriogonadotropin Receptor	Ss03384991_u1
MMP2	Matrix Metallopeptidase 2	Ss03394318_m1
PGR	Progesterone Receptor	Ss03374440_m1
PTGES	Prostaglandin E Synthase	Ss03392129_m1
PTGS2	Prostaglandin-Endoperoxide Synthase 2	Ss03394694_m1
STAR	Steroidogenic Acute Regulatory Protein	Ss03381250_u1
TF	Transferrin	Ss04955454_m1
TIMP1	Tissue Inhibitor Of Metalloproteinases 1	Ss03381944_u1
TNFα	Tumor Necrosis Factor-α	Ss03391318_g1
VIM	Vimentin	Ss04330801_gH
*ACTB**	Beta-Actin	Ss03376081_u1
*GAPDH**	Glyceraldehyde 3-phosphate Dehydrogenase	Ss03375435_u1
*HPRT1**	Hypoxanthine–guanine Phosphoribosyltransferase	Ss03388274_m1

*Reference genes
